# Who should mark the homework? Concussion, conflicts of interest, and the constitution of expertise

**DOI:** 10.1080/09581596.2025.2507854

**Published:** 2025-06-03

**Authors:** Gregory Hollin

**Affiliations:** Department of Sociological Studies, University of Sheffield, Sheffield, UK

**Keywords:** Concussion, conflicts of interest, dementia, expertise, sport

## Abstract

Concussion in sport is increasingly understood as a public health crisis. A key facet of this crisis concerns the claim that industry-funded research results in conflicts of interest that fundamentally compromise scholarship. This poses a particular problem for policymakers when adjudicating upon who counts as an expert and what to do with the evidence that they provide. In this paper, I explore these matters in relation to the ‘Concussion in Sport’ report produced by the UK’s House of Commons’s Digital, Culture, Media and Sport Committee. I ask, first, which stakeholders submit evidence to the Committee and, second, how evidence provided by those stakeholders is marshalled within the report itself. I show that, despite concerns about conflicts of interest, a significant body of interdisciplinary scholarship is submitted to the Committee. The report itself, however, understands academic scholarship as being both deficient and compromised, drawing exclusively upon epidemiological and neuroscientific work. I conclude by suggesting such an approach compromises the committee’s own hope for an increasingly expansive notion of expertise.

## Introduction

In the first decades of the twenty-first century, there has been a significant increase in discussion surrounding the links between head trauma and neurodegenerative disease (Livingston et al., 2020). This discussion has most frequently revolved around Chronic Traumatic Encephalopathy, or CTE, ‘a neurodegenerative disorder associated with brain trauma’ (Bieniek et al., 2021, p. 210). While numerous groups – including domestic abuse victims (Casper & O’Donnell, 2020) and military personnel (Priemer et al., 2022) – have been posited as being at risk for CTE, it is the risk associated with contact sports that has dominated attention (Mackay et al., 2019). The sum of these concerns about sport, brain health, and neurodegenerative disease is the so-called ‘concussion crisis’ of the present day (Malcolm, 2020b).

The argument that there are significant iatrogenic, neurological harms associated with sporting activity represents a profound questioning of a ‘sport-health ideology’ that posits sport to be unquestioningly beneficial for individual and public health (Malcolm, 2017). Indeed, within the popular press, the concussion crisis is frequently framed as both an ‘existential threat’ to sport (Fainaru-Wada & Fainaru, 2013, p. 6) and a ‘silent epidemic’ of disease that is about to ‘explode’ (Carroll & Rosner, 2012, p. xii). The fury elicited by the concussion crisis has been further heightened by allegations that sport’s governing bodies have engaged in a ‘cover-up and [the] falsification of research’ (Bell et al., 2019, p. 112). Undoubtedly, widely publicised incidents such as the publication of misleading injury statistics by World Rugby (Piggin & Pollock, 2017; Raftery, 2017), and the extensive research misconduct committed by one of the leading ‘sceptical’ authors in the field (Casper & Finkel, 2022, p. 1334; Macdonald et al., 2022) have significantly eroded trust in sport’s governance and speak to a broader crisis in (biomedical) expertise (Eyal, 2019; Kattumana et al., 2023).

In 2021, the United Kingdom’s House of Commons’s Digital, Culture, Media and Sport (DCMS) Committee convened in order to consider evidence relating to concussion in sport. It is clear that the pervasive mistrust, discussed above, was of central importance to these proceedings, with the chair of the DCMS hearings stating that he was ‘astounded that sport should be left…to ‘mark its own homework’ when it comes to the risks associated with traumatic brain injury (Reuters Staff, 2021). The report that the DCMS Committee would come to publish represents the most significant intervention from UK policymakers in this area, and continues to shape policy and policy-derived outputs in the UK (Research Forum, 2024; UK Government, 2023). Furthermore, the report represents a state-of-the-field summary that offers continuing insight into the imaginaries that shape ongoing understandings and policy proposals related to brain injury suffered whilst participating in sport.

In this paper, I explore the production of the DCMS Concussion in Sport report. Given that, as discussed above, conflicts of interest and the legitimacy of experts are central to the concussion crisis, I pay particular attention to, first, the stakeholders who feed into the knowledge production process and, second, how the evidence provided by these stakeholders is deployed within with the Concussion in Sport report itself. I show that a wide array of actors submit evidence to the DCMS Committee and that, whilst much of this evidence is produced by British authors writing on archetypally British sports, the submitted academic evidence constitutes a diverse and interdisciplinary body of work. Within the final report, however, academic scholarship is understood as fundamentally deficient and significantly compromised, with only epidemiological and neuroscientific work exempted from critique and included within policy documents. I conclude by discussing the potential implications of this rendering of academic knowledge.

## Concussion and crisis

A small but growing body of social scientific literature has sought to examine the sociocultural dimensions of the concussion crisis (Malcolm, 2020b; Ventresca & McDonald, 2020). These studies have considered concussion in the context of numerous sports, most prominently American football (e.g. Morrison, 2020; Rugg, 2020), soccer (e.g. Malcolm, 2020a), and rugby union (e.g. Malcolm, 2009).

As noted in the introduction, a consistent point of emphasis across both academic and popular literatures has been upon apparent conflicts of interest, and even conspiracy (Barr, 2020), in the context of brain injury. It has been widely suggested, for example, that ‘sport looks at concussion like big tobacco sees smoking’ (Convery, 2023 see also:; Bell et al., 2019, Chapter 2; Bachynski & Goldberg, 2018) with sport governing bodies argued to be deliberately obfuscating the links between brain injury and neurodegenerative disease (Endedijk & van Steenbergen, 2020; Fainaru-Wada & Fainaru, 2013). Such obfuscation is argued to come, in part, through industry-sponsored research that is alleged to result in biased conclusions (Bachynski & Goldberg, 2018; Partridge & Hall, 2014). It has also been argued that team physicians may be conflicted, torn between player health and team performance (Baugh et al., 2020; Partridge, 2014). In this context, the concussion crisis is multidimensional (Kattumana et al., 2023): not only a public health crisis (Bell et al., 2019; Malcolm, 2020b, Chapter 7), but part of an ongoing of crisis of expertise (Eyal, 2019) wherein those believed to be responsible for both understanding brain injury and prioritising player welfare have failed in their duties.

Foregrounding this crisis in expertise emphasises two aspects of the concussion crisis. First, understandings of concussion become anchored to our current moment wherein crisis talk is ‘dominant in our media coverage’ (Masco, 2017, p. s65) and the self-evident legitimacy of experts cannot be assumed (Eyal, 2019; Gauchat, 2023). Second, orienting towards a crisis in expertise highlights a particular challenge facing policymakers who, in the process of enacting evidence-based policy, must ask ‘who counts as an expert given alleged conflicts of interest?’; ‘what does one do with existing evidence?’; and, ‘how does one imagine future research?’

Amidst this uncertainty, various paths forward present themselves to government and public health officials. One option would be to draw ‘on a wider range of evidence about health and wellbeing’ (Green et al., 2022, p. 596), combating conflicts by pluralising and triangulating expertise. A second approach would involve retrenchment and the consecration of existing knowledge hierarchies – an attempt to sure up knowledge by hearing fewer and fewer voices. It goes without saying that the politics, ethics, and epistemics of these approaches differ radically.

This is the orientation that I bring to an analysis of the 2021 DCMS hearings and Concussion in Sport report. In this article, I examine the constitution of expertise – primarily, but not exclusively, academic expertise – within the DCMS hearings and report. I consider, first, the stakeholders who claim expertise into the topic at hand and, second, how that expertise is marshalled within the report itself.

## Methods

### Materials

Materials for this analysis derive from three primary sources, all of which relate to the Concussion in Sport report.

**Figure 1.**
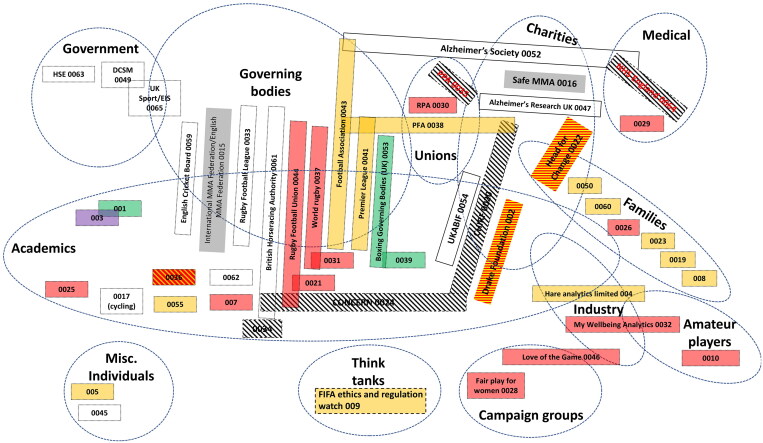
An illustrative social/world arena map. In this map, colours denote the sport with which a submission is primarily concerned (e.g. yellow = soccer; red = rugby union). Rectangular boxes represent a submission to the DCMS Committee, oval boxes represent social worlds. Overlaps represent an attempt to map porosity between worlds/submissions. This map is for written evidence submitted to the DCMS and should be read heuristically.

First, I draw upon written evidence that was submitted to the DCMS Committee (see Figure 1 and Table 2). Fifty-one written submissions have been published on the DCMS website, of which 47 were published on the 20 April 2021.^1^ Two additional submissions were published in the following weeks, while two ‘supplementary’ submissions, both from parties who gave oral evidence, were submitted in July of the same year. Each of these submissions was published with a unique identifier (e.g. CON0030) which will be used throughout this article. These submissions ranged in length from a few sentences to over 20 pages but, in sum, constitute 250 pages of evidence. As will be discussed in greater detail below, these submissions were made by a range of different actors, including academics, charities, sport governing bodies, and family members affected by concussions and their long-term consequences.

Second, I utilise oral evidence given to the DCMS Committee. The committee met over five days in March, April and May 2021. Over those five days, the committee convened ten panels featuring 27 witnesses, with each panel representing a particular area of expertise (see Table 1). While several witnesses could easily have slotted into a different panel, these witnesses appeared present in a particular capacity (as ex-players or charity representatives, for example). In sum, the committee asked witnesses 503 questions, which resulted in approximately 200 pages of transcribed evidence.

**Table 1.** Oral witnesses at the DCMS concussion in sport sessions in March, April, and May 2021. Representative affiliations are as listed in the DCMS report. Panel names are my own and should be read heuristically.

Date	Panel	Representatives
9 March	1: Scientists	Willie Stewart (University of Glasgow)
		Craig Ritchie (University of Edinburgh)
	2: Brain Charities	Michael Grey (UK Acquired Brain Injury Forum)
		Peter McCabe (Headway)
		Richard Oakley (Alzheimer’s Society)
23 March	3: Players	Monica Petrosino (TeamGB ice hockey)
		Eleanor Furneaux (TeamGB skeleton bobsled)
	4: Player Charities	Dawn Astle (Jeff Astle Foundation)
		Chris Sutton (Jeff Astle Foundation)
		Kyran Bracken (Progressive Rugby)
		John Fairclough (Progressive Rugby)
	5: Governing bodies	Charlotte Cowie (Chief Medical Officer, Football Association)
		Éanna Falvey (Chief Medical Officer, World Rugby)
		Mike Loosemore (Chief Medical Officer, TeamGB Boxing and TeamGB Snowsports)
		Bill Sweeney (Chief Executive, Rugby Football Union)
27 April	6: Players’ unions	Damian Hopley (Chief Executive, Rugby Players Association)
		Paul Struthers (Director, Professional Players Association)
		Gordon Taylor (Chief Executive, Professional Footballers Association)
	7: Sporting institutes (1)	John Etherington (Medical Director and consultant rheumatologist, Faculty of Sport and Exercise Medicine UK)
		Richard Sylvester (consultant neurologist, Institute of Sport, Exercise and Health)
18 May	8: NHS	Alistair Burns (National Clinical Director for Dementia and Older People’s Mental Health, NHS England)
	9: Sporting institutes (2)	Niall Elliott (Head of Sports Medicine, Sport Scotland)
		Rod Jaques (Director of Medical Services, English Institute of Sport)
		Sally Munday (Chief Executive, UK Sport)
		Phil Smith (Director of Sport, Sport England)
25 May	10: State actors	Nigel Hudson (Minister for Sport and Tourism, DCMS)
		Ben Dean (Director, Sport, Gambling and Ceremonials, DCMS)

Finally, third, is the Concussion in Sport report itself. The report is authored by the DCMS Committee, chaired by Julian Knight (Conservative) and whose membership consisted of 11 Members of Parliament (6 Conservative; 4 Labour; 1 Scottish National Party). All members attended at least some of the oral evidence sessions. The report itself is 38 pages in length.

### Methods

There are two primary goals of this analysis. The first is to examine the constitution of expertise in the context of sports-related concussion and the concussion crisis more broadly. The written and oral evidence submitted to the committee is most relevant to this goal. The second is to see how evidence provided by those experts is marshalled and deployed within governmental reports: unsurprisingly, the Concussion in Sport report is most relevant here.

In order to examine the constitution of disciplinary expertise, I relied primarily upon a process of ‘social worlds/arenas mapping,’ as elucidated by Clarke (2005). For Clarke, the purpose of these ‘cartographic exercises’ (Gagnon et al., 2010, p. 248) is to make ‘*collective* sociological sense’ (Clarke, 2005, p. 110 italics in original) out of the ‘situation.’ For Clarke, the ‘arena’ is a particular space (a hospital, for example), while the social worlds are collectives that speak to this arena (for a hospital, these might include pharmaceutical companies, nurses, medical technology industries and so forth – Clarke, 2005, p.118).

For present purposes, the DCMS hearings constitute the arena, whilst the stakeholder groups represent social worlds. The social worlds are mapped out onto the arena (see Figure 1), with dotted lines and overlaps representing porosity between social worlds. (In Figure 1, for example, ‘families’ are often representatives of ‘charities’ and so those social worlds overlap on the map.) For this project, I am primarily interested in stakeholder groups that relate to different facets of life (family, academic, charity) and so have represented these worlds spatially. With submissions from academics, I also grouped submissions arising from the same discipline together. Accordingly, submissions from academics working within, for instance, law schools were placed next to each other.

It is worth being clear about what can, and cannot, be represented within a world/arena map. First, there are clear limitations to how much of a densely connected, always in flux, social world can be represented on a two-dimensional, static map: some connections will always be missed, others given undue salience, within a single map. Second, it would be possible to produce very different maps based on the same information. For example, an arena mapped spatially on the basis of affiliation to different sports would look very different to that in Figure 1. In sum, then, world/arena maps should be read heuristically and as a research aid, rather than a research output.

### Ethics

The larger study from which project arises has been reviewed and given a favourable opinion by an ethics committee at The University of Sheffield (reference 045515).

## Analysis

### Written submissions

The written submissions represent a particularly interesting sample of documents produced by a range of individual and institutional actors sufficiently invested in sports-related concussions to write to a parliamentary committee. As can be seen in Table 2, 1 identified 12 social worlds within the written submissions. The most populous social world was ‘academics,’ with 13 submissions, followed by ‘governing bodies’ with 9. The boundaries between these social worlds are, frequently, porous: academics, for example, declare that they are working with charities, governing bodies, and industry; charities with governing bodies, medical bodies, and families. Thus, and as is the case with other diseases, concussion in sport appears as ‘a matter of concern’ for an array of actors, many of whom ‘co-exist and interfere’ with each other (Moser, 2008, p. 104).

**Table 2.** Social worlds identified in the written submissions. Social world names are my own and should be read heuristically.

Social world	Number of submissions	Example
Academics	13	Senior lecturer in sport policy CON0007
Amateur players	1	Amateur rugby player CON0010
Campaign groups	2	‘Love of the Game’ CON0046
Charities	7	‘Alzheimer’s Society’ CON0052
Families	6	Daughter of parent with dementia CON0023
Governing bodies	9	Football Association CON0043
Industry	2	Analytics company CON0032
Medical	2	Director at NHS England CON0064
Miscellaneous Individuals	2	Submission about health and safety law CON0045
Sporting Unions	3	Professional Footballers’ Association CON0038
State actors	3	Health and Safety Executive CON0063
Think tanks	1	FIFA Ethics and Regulation Watch CON0009

In terms of substance, these submissions are dominated by those dealing with soccer (17) and rugby union (15). The only other sports of primary concern in more than one submission are boxing and mixed-martial arts (MMA), for which there are three and two submissions respectfully. Thus, despite both the wide array of actors producing written submissions and the diversity of sports wherein concussion is potentially relevant, there is a congealing here around two prominent, archetypally British (Gilroy, 2001), sports. Closer inspection reveals this weight of emphasis to come from what might crudely be called ‘social’ actors: Of the 14 submissions made by amateur players/campaign groups/families/industry/miscellaneous individuals/think tanks, all except one concern rugby union or soccer. Indeed, of the six submissions coded as ‘family’, five are written by daughters of ex-professional soccer players with dementia, while submissions from both amateur players and campaigns, whilst small in number, exclusively concern rugby union. By comparison, the 9 submissions from governing bodies include those associated with boxing, cricket, horse racing, MMA, rugby league, rugby union, and soccer; submissions from both state actors and charities are frequently generalist or multi-sport.

Written submissions were provided by academics working within a number of different, and quite diverse, disciplines. While both of the academics invited to give oral evidence to the committee are brain scientists, amongst the 13 written pieces submitted by academics there are contributions from scholars in engineering, health science, law, maths, sociology, and sports science, as well as the neurosciences. These are submissions that concern everything from the development of biomarkers (CON0024); to the difficulty of pitch-side diagnosis (CON0036); to the effects of litigation upon volunteer numbers (CON0001); and the forces experienced while heading soccer balls made of different materials (CON0055). These scholars also write on a wide-range of sports: whilst six submissions deal predominantly with soccer and/or rugby union, others consider American football, boxing (2), cycling, or are extremely generalist/multi-sport in orientation. In sum, 30 academics, working in 14 institutions, are named as authors across these publications. The institution with the most submissions (4) and named authors (7) is Loughborough, followed by Exeter (1 submission, 5 authors). All lead authors are based in the UK, with one signatory located in the United States and one in Australia.

Before moving on, it is worth pausing briefly to note possible stakeholders who are absent from the arena (Washburn, 2013, p. 456). Firstly, and in the written submissions, there is comparatively little input from those who actively participate in sport. There are no submissions from professional players and a lone submission from a former amateur rugby player (CON0010). There are no submissions from coaches, referees, parents, or other club officials. Second, while sports-related concussions are highly mediatized (Bell et al., 2019; Ventresca, 2019), with significant coverage in British newspapers such as the *Daily Mail* and the *Guardian*, there are submissions from neither news organizations nor individual journalists (and no journalists gave oral evidence). Finally, almost all the submissions are from individuals and organizations based in the UK. The Dublin-based World Rugby, for example, is the only international governing body to submit evidence.

In sum, then, the concussion crisis, as depicted within submissions to the DCMS Committee, is rendered in starkly nationalistic terms: the overwhelming majority of submissions are from stakeholders based in the United Kingdom, whilst the majority of evidence concerns the archetypally British sports of rugby union and soccer. That said, there is a broad base of expertise for the DCMS to draw upon. A wide range of stakeholders submitted evidence, with an institutionally and disciplinarily diverse body of academic work constituting the largest social world.

### The concussion in sport report

If the above subsection deployed social worlds/arena mapping in order to get a grip on which actors engage with matters of sports-related concussion, this second section turns instead to examine how those contributions are taken up within the written report. How does evidence travel, and which sorts of evidence get transformed into policy recommendations?

The authors of the Concussion in Sport report describe themselves as engaging in ‘evidence-based policy’ (p.6), and the report leans heavily on published, written, and oral evidence, with over 120 citations across fewer than 30 summary pages. Written evidence submitted to the committee features prominently within the report, with 33 citations in total. Six out of seven submissions from charities are cited; 4/6 from families; 3/3 from government; and 4/9 from governing bodies. With specific regards given to the citation of academic work, however, uptake is comparatively sparse with only 4 out of 13 submitted pieces being cited. Furthermore, each of these pieces is cited just once. In these crude terms at least, the academic social world diminishes in significance in the final written report: while just shy of 30% of all written submissions to the committee came from academics, these sources represent around ten percent of the citations within the final DCMS report.

Just as important as the question of *what* work gets cited is the question of *how* that work gets cited. The first three citations to written submissions from academics are, unsurprisingly, in a portion of the report entitled ‘research activity’. The first piece of written evidence to be cited in the DCMS report, CON0039, was submitted by neurologists, although the piece is cited to make a more general point:

‘It is indubitable however that there is a greater demand for funds than there is money available, and a number of written submissions outline the list of unknowns that the right research projects might begin to address’ (cited on p.25)

A second submission (CON0031), from a sports scientist, makes much the same point as above and is quoted at length:

‘We have found it challenging to secure funding for research in this field. Few grant calls are open to concussion research, particularly in sport. To date, most of our research has been conducted with no, or very little external funding.’ (quoted on p.25)

While it will not be a surprise to this audience to find academics bemoaning a paucity of research funds, a third submission (CON0025) is cited to make an even more damning point:

‘More pointed criticisms of the state of research in this area were made by academics from Newcastle University, who said that “It is striking that in the UK there is almost no independent research into rugby injuries, research is almost exclusively funded by the rugby unions”…’ (quoted on p.25)

This argument may be correct as it pertains to biomedical research, but written submissions demonstrate a significant body of interdisciplinary research on concussion in sport, much of which declares no relationship with governing bodies. This body of interdisciplinary scholarship is ignored with the final report, however, with evidence such as that quoted above marshalled instead into something akin to an ‘anti-epistemology’ (Galison, 2004) and used to make a two negative and related points: there is not enough funded research taking place, and research that is funded is significantly compromised. This understanding of academic research as being, first, exclusively biomedical in nature and, second, fundamentally flawed, may explain the paucity of academic citations within the report: this is simply not the place from which reliable evidence arises.

Indeed, the deployment of these three quotes aligns neatly with the report’s understanding of research *tout court*, with two salient issues presented throughout the report: first, the state of the science is very uncertain. The report describes how there is ‘uncertainty in the science… information is often slow, and our current scientific knowledge does not demonstrate a causal link between particular sporting activities and later development of dementia’ (p. 5), there is a ‘lack of information about the scale of the problem’ (p. 7) and a ‘need for more conclusive evidence’ (p. 24). Second, there is a focus upon the issue of bias and poor-quality research. It is argued that methods are poor; that publication practices are poor; that sample sizes are poor. At least some of this inadequacy is, it is suggested, the direct result of bias that results from funding being funnelled through governing bodies (pp. 24–25). This claim is again indicative of how ‘research’ is imagined within the DCMS report, for while these are certainly concerns raised with regards to biomedical science, they are rarely present in the context of social scientific and humanities scholarship pertaining to brain injury.

A final, fourth, submission (CON0062) is cited in a separate section of the report entitled ‘the government’s role in regulating sport’. This submission, also from a sports scientist, is cited to critique a lack of government action following a previous report into medical provision in sport:

‘This working group [established after a 2002 report], of which I was a member and co-author…set a path forward… but the government failed to act…’ (quoted on p. 28)

This quote is used to evidence a more general point that ‘there is a history of the Government looking into issues of sporting safety and failing to follow through with practical interventions that make a difference’ (p. 28). As with the above citations about research funding, therefore, this submission is not rendered as a positive contribution to knowledge production but is, instead, used to illustrate a negative, to demonstrate that something has gone awry.

As is commonly the case with parliamentary hearings (Ray et al., 2022), evidence provided orally by academics is centralised within the report, with evidence given by Willie Stewart, a prominent neuropathologist from the University of Glasgow, particularly central. Indeed, the ‘FIELD Study,’ for which Stewart is the primary investigator, is cited more frequently than all of the written submissions in total. Despite the fact that it was funded by both the Football Association and the Professional Footballers’ Association, the FIELD Study is exempted from the aforementioned critiques of academic research, and is described as having ‘established a strong correlation between a career in football and a significantly increased incidence of dementia in later life’ (p.6); later the study is described as ‘solid scientific evidence’ (p. 18), with specific epidemiologic risk factors being quoted.

A curious aspect of the final DCMS report is that it draws significantly upon neuroscientists’ oral evidence even when that evidence concerns topics quite some distance from epidemiology or brain science. As an example, Stewart is quoted in the final report as saying that ‘I know a lot of pitch-side medical doctors… I have not met one yet who strikes me as conflicted by their role,’ (p. 14). Stewart is cited an additional 3 times when describing the difficulties these medics face. It is worth noting here that the committee received written evidence (CON0036) from a sociologist of sport who has examined this topic for 20 years, who cites a significant body of work published in peer-reviewed journals, and who complicates the picture presented in the report -- this work is left uncited.

Finally, and while it is not central to the report or its recommendations, it is perhaps worth noting how the DCMS report envisages the future of scientific research. The only reference to future research comes in the form of an assertion that there ‘is a need to understand the mechanisms by which neurological disease occurs to allow for the development of treatments that might mitigate the severity of disease or even prevent it happening at all’ (p. 24). Following the publication of DCMS report, the UK Government announced a ‘Sports Concussion Research Forum’ that was intended to address this need and ‘identify common research goals and priorities across the sector’ (Minister for Sport, Tourism, Heritage and Civil Society, 2021, p. 26). With the exception of a public health epidemiologist, academic membership of this forum consists near-exclusively of biomedical researchers who, in turn, identify what appear to be near-exclusively biomedical research priorities (Research Forum, 2024). Contra to the stated aims of the DCMS report, the membership of this forum is potentially more epistemically conservative than the much criticised International Consensus on Concussion in Sport group, which has gone someway to incorporating diverse disciplinary perspectives (Schneider et al., 2023). In sum, from both the report and its fallout, it seems clear that it is biomedical – and particularly neuroscientific – research that is able to resolve the concussion crisis.

## Discussion and conclusion

The UK Government responded to the Concussion in Sport report in December 2021, formally noting it’s agreement with many, although certainly not all, of the recommendations contained therein (Minister for Sport, Tourism, Heritage and Civil Society, 2021). Perhaps the most notable outcome has been the production of standardized, UK-wide, concussion guidelines for grassroots sport (UK Government, 2023) – the development of which were led by ‘dementia care’ expert Laurence Gellar, who was appointed as a ministerial advisor on concussion in sport in 2021 (House of Commons, 2021; Andrew & Department for Culture, Media and Sport, 2023; UK Government, 2023). These guidelines certainly represent a positive contribution to knowledge but, as discussed above, the DCMS report can also be understood as manifesting the ongoing crisis in expertise (Eyal, 2019). The committee raise concerns about the quantity and quality of research, the possibility of interference from sport’s governing bodies, and the vast number of unknowns that need to be addressed through rigorous, impartial research.

Within the self-contained arena that constitutes the DCMS Concussion in Sport hearings this ‘anti-epistemology’ (Galison, 2004) is predicated upon the exclusion of a fairly substantial body of interdisciplinary research: I discussed a wide range of submissions, including 13 authored by 30 academics working across 14 institutions, at least 7 disciplines, and concerning a wide array of topics. The positive contributions to knowledge offered by these stakeholders are entirely ignored, with the only certitudes arising from epidemiological research, most notably the FIELD Study, and imagined future neuroscience. Strikingly, the DCMS Committee appear sufficiently uninterested in all non-biomedical research that such claims are either treated as irrelevant; self-evident truisms; or, alternatively, as amenable to investigation by non-specialists.

The Concussion in Sport report is thus strangely bifurcatory. On the one hand, the report advocates for a significant epistemic reordering wherein a precautionary approach takes precedent over a fixation on certainty (paragraph 39–40), and where expertise is radically rearticulated so as to include not only scientists, but sporting institutes and campaign organizations (paragraph 70). On the other, the committee entirely reinforce the status quo: inviting only brain scientists, positively citing only biomedical research, envisaging progress only in terms of basic neuroscience which enables the development of new treatments. The theory is an expansive form of expertise; the reality appears brain-centric, dominated by natural scientists.

There are, I suggest, at least two consequences of this framing. The first is that the history of Alzheimer’s research should give pause if the hope is for a biomedical magic bullet, for the search for diagnostics and cures in that area has been costly, fraught, and frequently disappointing. The second is that it paints a bleaker picture than is necessarily the case. Written submissions to the panel weave a tapestry of interdisciplinary research, with contributions emanating from engineering; law; mathematics; and the health, sport, and social sciences. This is scholarship that concerns not only biomarkers, but pitch-side diagnosis, the effects of litigation upon volunteering, and the effects associated with heading soccer balls made of different materials. It is, in short, scholarship that addresses many of the questions that the DCMS sought to find.

## Data Availability

This research has been undertaken with the following publicly available data sets: https://committees.parliament.uk/work/977/default/publications/written-evidence/?page=1; https://committees.parliament.uk/work/977/default/publications/oral-evidence/; https://committees.parliament.uk/work/977/concussion-in-sport/publications

## Note

1 Putting these 51 submissions into some context: In the 2021-22 session, the DCMS Committee produced 12 reports, of which 7 appeared to incorporate written submissions. These seven inquiries received between 31 and 214 written statements, with those based around sport receiving 31 (‘major cultural and sporting events’), 51 (‘concussion in sport’), and 108 (‘sport in our communities’) responses. Full inquiry details can be found: https://committees.parliament.uk/committee/378/culture-media-and-sport-committee/work/inquiries/?status=any&page=2.
